# Dried Spot Paradigm: Problems and Prospects in Proteomics

**DOI:** 10.3390/ijms26083857

**Published:** 2025-04-18

**Authors:** Olga I. Kiseleva, Yuriy A. Ikhalaynen, Ilya Y. Kurbatov, Viktoriia A. Arzumanian, Polina A. Kryukova, Ekaterina V. Poverennaya

**Affiliations:** Institute of Biomedical Chemistry, Pogodinskaya Street, 10, 119121 Moscow, Russia; olly.kiseleva@gmail.com (O.I.K.); ikh.ya@yandex.ru (Y.A.I.); kurbatild@gmail.com (I.Y.K.); arzumanian.victoria@gmail.com (V.A.A.); polinakpa@gmail.com (P.A.K.)

**Keywords:** dried sample, proteomics, biomarker, diagnostics

## Abstract

The analysis of biological fluids plays a crucial role in biomarker discovery, disease diagnostics, and precision medicine. Dried sample carriers—such as dried blood spots, dried plasma, serum, saliva, tears, and urine—have emerged as powerful tools, offering advantages in sample collection, storage, and transport, particularly in remote and resource-limited settings. Recent advances in proteomic methodologies have expanded the potential of these dried matrices, yet challenges related to protein stability, sensitivity, and standardization persist. This review critically examines the current state of proteomic investigations using dried biological fluids. Furthermore, we compare proteomics’ progress in this field with other omics approaches, such as metabolomics, to contextualize its development and integration potential. While dried fluid proteomics is promising for non-invasive diagnostics and large-scale epidemiological studies, addressing technical limitations will be essential for its broader adoption in clinical and translational research.

## 1. Dried Matrices: Long Story Short

**Figure 1:**
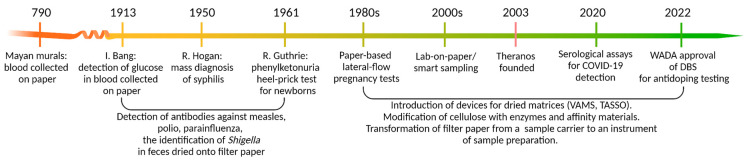
A chronological journey through key innovations in paper-based diagnostics, from the early use of DBSs to cutting-edge smart sampling techniques. The figure highlights Ivar Bang’s pioneering work in glucose detection [1], Guthrie’s breakthrough in newborn screening for PKU [2], and the introduction of lateral-flow pregnancy tests [3]. Advancements in VAMS and functionalized substrates further refined biomarker detection [4,5,6,7]. Recent developments in paper-based systems continue to expand their role in personalized medicine and large-scale health monitoring [8,9,10].

The idea of using paper in the clinical analysis of biologically relevant substances is not new (Figure 1). Isolated historical evidence suggests that blood drops were collected on paper as part of sacrificial rituals as early as 780 AD, as depicted in Mayan murals [1].

More than eleven centuries later, in 1913, Ivar Bang published his observations on using dried blood spots (DBSs) to determine glucose levels in blood droplets applied to washed filter paper, thus laying the foundation for modern clinical microchemistry. This discovery triggered a series of diverse research efforts, the most notable achievement of which was the implementation of population-wide epidemiological surveys. These advances enabled large-scale screening programs for syphilis, measles, polio, and even dysentery—though in the latter case, the samples were not blood but feces dried onto filter paper and shipped via regular mail from Indonesia to The Netherlands. Another milestone in the history of biomaterial-based diagnostics was the development of a method for detecting phenylketonuria (PKU) from DBSs obtained via heel prick in newborns, introduced by R. Guthrie in 1961. A dried disc punch was incubated on a Petri dish seeded with Bacillus subtilis in the presence of the growth inhibitor B-2-thienyl-alanine. High phenylalanine concentrations in the blood sample counteracted the inhibitory effect, facilitating bacterial growth around the disc, which served as a diagnostic marker for PKU.

Throughout his career, Guthrie played a pivotal role in expanding newborn screening beyond PKU to include a wide range of metabolic disorders and endocrine conditions [2]. Today, newborn DBS screening has been further extended to detect conditions such as cystic fibrosis, severe combined immunodeficiencies, spinal muscular atrophy, etc. Although more precise and efficient modern techniques have replaced Guthrie’s original bacterial inhibition PKU assay, the core principle remains unchanged—all these tests still rely on blood samples collected on filter paper.

Despite initial resistance from leading medical professionals and organizations, Guthrie successfully advocated for the widespread implementation of mandatory newborn screening, which now represents a “modus operandi” across much of the developed world. The 1980s marked a breakthrough in accessible diagnostics with the advent of at-home lateral-flow pregnancy tests, enabled by advances in antibody technology and human chorionic gonadotropin detection. Their success stemmed from the reliable results, test simplicity, and equipment-free use [3].

The 2000s saw the rise in smart sampling—transforming paper substrates into active tools for early sample preparation [4]. Notable examples include carriers pre-loaded with proteolytic enzymes or antibodies, significantly reducing in-lab processing time while enabling protein detection at low ng/mL levels. Plasma separation cards, employing multi-layer absorption and filtration, further advance this concept by converting whole blood into a dried plasma-like specimen without centrifugation [5,6,7].

Alongside advances in cellulose design, volumetric devices such as volumetric absorptive microsamplers (VAMS) gained traction, offering improved quantification through precise sample volume control. This approach enhances extraction yield and reproducibility while minimizing hematocrit-related variability. Beyond traditional clinical diagnostics [8], VAMS has been approved for anti-doping surveillance [9] and screening programs during the COVID-19 pandemic [10].

The downfall of the too-good-to-be-true company Theranos—which tarnished the concept of decentralized medicine [11]—has not hindered the advancement of patient-centered solutions; instead, it has catalyzed further innovation. Dry matrices are increasingly gaining traction, fueled by the rising interest in patient-centric medicine. The minimally invasive collection of dried blood spots, a practice long established in neonatal and pediatric diagnostics, is esteemed for its reliability and the ability to transport samples without cold chain logistics. For instance, the World Health Organization advocates using dried blood spots in broad screening initiatives for infectious diseases such as hepatitis B, C, and human immunodeficiency virus in regions with limited healthcare resources [12]. With advancements in high-throughput technologies, biological matrices applied to solid supports have evolved beyond traditional tests to diagnose specific diseases through single markers, as exemplified by the classic PKU or HIV tests.

In this review, we highlight recent progress and emerging challenges associated with the application of omics technologies to biomaterials preserved on solid supports, with a particular emphasis on clinically relevant proteomic studies. To provide a comprehensive overview, we compare the achievements and unresolved issues in the proteomic profiling of dried spots of various biofluids against the backdrop of their molecular characterization across other omics subdisciplines.

## 2. Types of Biological Liquids to Apply

The simplicity and directness of depositing biological fluids onto a solid carrier predictably spurred efforts to adapt classical analytical workflows to the dry spot paradigm. In the following subsections, we will examine the progress in proteomics applied to diverse dried matrices compared to other “omics” disciplines.

### 2.1. Blood and Its Components

Blood is the most popular biomaterial for proteome analysis using dry spot methods. The blood collection process usually involves a simple finger prick, reducing patient discomfort and allowing for repeat sampling even in pediatric populations. After collection, the blood is dried to form a stain characterized by remarkable stability under environmental conditions [13]. Drying stabilizes proteins [14], preserving a wide range of components, including high-abundance ones such as albumin and immunoglobulins, and biomarkers in lower concentrations critical for early disease detection [13]. The correlation between liquid and dried samples is high (0.97) [15]; however, despite the overall stabilizing effect, drying can slightly but consistently affect protein identification by inducing aggregation, disulfide bond formation, oxidation, and deamidation [14,16].

The potential of a dried blood spot (DBS) as a sample type has been well-demonstrated in studies where mass-spectrometry-based methods have consistently detected hundreds to nearly two thousand proteins in a single sample, among which five hundred and eighty-five are disease-related proteins cataloged in the Online Mendelian Inheritance in Man (OMIM). This finding indicates that DBSs can serve as an equally valuable source of proteomic information compared to whole blood or plasma in the conventional liquid form [17]. By comparison, human plasma—which is arguably the most extensively studied biological fluid in proteomics—has approximately 3500 proteins detected, according to the Human Proteome Organization’s (HUPO) Human Plasma Proteome Project (HPPP). This number is based on an analysis of nearly 180 studies with a stringent protein-level false discovery rate of 1% [18].

Numerous examples exist of proteomic biomarkers derived from DBSs used in clinical diagnosis [19,20]. For example, DBS has long been used in newborn screening programs for metabolic disorders. More recently, it has been adapted to measure specific protein markers—such as ATP7B for Wilson’s disease screening and panels of proteins associated with primary immunodeficiency—to improve diagnostic accuracy and monitor patients [21]. The utility of DBS was also demonstrated in assessing health status during the COVID-19 pandemic, where the authors conducted in-depth proteomic profiling of DBS samples to obtain clinically relevant insights for monitoring disease progression [22].

One of the key factors limiting the potential of proteomic studies using DBS is the influence of variability in hematocrit (HCT) levels, defined as the ratio of red blood cell volume to total blood volume. Variations in HCT levels lead to variations in stain size: blood samples with high HCT produce smaller spots, while those with low HCT generate larger ones [23]. This variability causes uneven protein distribution on the filter paper, reducing the accuracy of subsequent sampling and potentially leading to inaccurate quantitative results [24]. The impact is particularly pronounced when calibration control blood spots differ in HCT from experimental samples [23]. Fluctuations in HCT levels can arise from individual physiological characteristics (such as age, sex, and race), pregnancy status, medical conditions (e.g., anemia or polycythemia), as well as lifestyle factors (including smoking habits and dietary patterns). Such lifestyle-related factors are particularly challenging to monitor and correct computationally [24].

VAMS (volumetric absorptive microsampling) is the blood-collecting method that overcomes the problem of hematocrit fluctuations by the collection of an accurate volume of blood (e.g., 10 μL) into a porous substrate [23].

Another crucial factor to be considered when exploring the potential of DBS in the context of fundamental proteome research and clinical applications is the stability of proteins contained in DBS, depending on storage conditions. Studies commonly demonstrate that proteins in DBS remain more stable when stored at lower temperatures. For example, at the temperature of −24 °C, a set of oncomarkers retained detectability for up to 30 years, which was confirmed with PEA (proximity extension assay) [25]. After 10 years of storage, the content of proteins preserved at +4 °C decreased to 80%, whereas at −24 °C it only declined to 93%, which evidences the advantage of deep freezing. Similarly, other MS-based experiments with various timeframes and sets of temperatures support this idea: 35- and 154-day storage periods at −20 °C and +4 °C outperform +25 °C or +37 °C in protein detectability [26,27].

The vulnerability to temperature also varies between the analyte classes: for example, SARS-CoV-2 antibodies in DBS remain stable at room temperature in the short term and for up to two years at −20 °C, withstanding repeated freeze–thaw cycles [28,29]. In contrast, cytokines demonstrate significant losses at room temperature (13 out of 21 analytes degrade in 5 months) but storage at +4 °C or lower minimizes these changes [30].

The same conclusions can be applied to VAMS; for this method, ambient temperature is commonly supposed to be the gold standard for storage. However, 30 days of storage of VAMS samples in this condition causes analyte loss of less than 14% [31]. Another experiment on cytokine quantification after 5 months of storage discovered that samples kept at RT show the poorest results compared to +4 °C and −20 °C. Intriguingly, the best storage temperature differed among specific analytes [30].

Thus, to maximize the integrity of a broad spectrum of molecules, it is recommended that samples be stored at temperatures from −20 °C to −80 °C, humidity-controlled, and direct light and abrupt temperature changes avoided. Based on standardized protocols, these measures are critical for ensuring data representativeness in biobanks and long-term studies [32].

A few analytes perform poorly regardless of storage conditions, possibly due to inefficient recovery during extraction [31]. Extracting analytes from cards subjected to long-term storage may pose challenges. However, optimizing conditions and using isotopically labeled standards enables reproducible and quantitative determination of protein content from DBS [33].

Despite the extensive literature examining molecular profiles in DBS, research on dried plasma or serum remains strikingly limited. This disparity may stem from pragmatic considerations. Plasma and serum spot methodologies face significant competition from conventional liquid-based assays, which are already standardized and widely adopted in clinical laboratories. The biopharmaceutical industry and regulatory authorities have prioritized investment in DBS due to its proven utility in drug development and global health initiatives, where its logistical advantages—such as simplified storage and transport—are paramount. Consequently, innovations in dried plasma or serum techniques are often considered redundant unless they offer unique benefits over established DBS or liquid approaches.

While dried plasma (DPS) or serum spot (DSeS) could theoretically mitigate challenges associated with hematocrit variability and cellular interference, their practical implementation is hindered by notable limitations. These include complex collection protocols requiring anticoagulants or centrifugation, instability during long-term storage, and a lack of robust validation frameworks. Such drawbacks have curtailed their adoption, leaving DBS as the preferred methodology due to its operational simplicity, cost-efficiency, and adaptability across diverse settings.

The limited number of studies utilizing dried serum spots focuses on the analysis of low-molecular-weight substances: vitamins [34,35], mycotoxins [36], and pharmaceutical compounds [37]. Besides “metabolomic” applications, there is a significant corpus of ELISA-based projects [38] for detecting various infections in DSeS [39,40,41,42,43]. Despite the availability of such analysis, there are no reliable data on the application of comprehensive proteomic profiling of DSeS for clinical purposes via mass spectrometry [42,44]. Another avenue of protein analysis involves the quantification of ferritin [45]. Given that classical clinical assays measure ferritin in blood serum, DSeS appears promising for population-level screening of ferritin levels, particularly in tandem measurements of ferritin and transferrin receptors [46,47,48].

Almost simultaneously with the investigation of the applicability of DSeS for analyzing ferritin levels in the body (1998), the potential use of DPS was evaluated in 1999 [49]. A promising result for ferritin assessment using capillary blood was demonstrated with a modified DPS format known as the NoviplexTM Plasma Prep Card [50]. The key advantage of this method lies in eliminating preliminary blood sample preparation as the plasma separation is achieved through a removable filter that absorbs blood cells [51]. The applicability of this modification has been demonstrated not only in metabolic analyses [52] but also in targeted [51,53] and panoramic proteomic studies [54]. A similar type of DPS based on filtration principles, known as plasma separation cards, was developed by Roche [55]. These cards are primarily focused on applications in viral load testing [5,56] and serological studies [57,58], owing to their demonstrated increased sensitivity compared to dried blood spots (DBSs) at a comparable cost [55,59].

It should be noted that, despite the abundance of proteomic studies utilizing DBS, the body of research on the proteome of dried plasma and serum remains limited. Nevertheless, several notable proteomic investigations have focused on liquid components of blood dried on cellulose-based matrices. One study examines neurofilament light chain (Nf-L) in DPS as a potential early diagnostic marker for neurodegenerative diseases [60]. In this work, Nf-L levels in DBS and DPS were compared using electrochemiluminescence, with adjustments made for HCT influence. Another study of practical significance explores the application of dried serum spots for diagnosing intrauterine growth restriction (IUGR), a pathological pregnancy condition characterized by the fetus failing to achieve its genetically determined growth potential. This research highlights the potential of dried serum-based methods in identifying biomarkers associated with IUGR, underscoring their clinical relevance [61]. A noteworthy publication [40] describes the application of a precise volume of blood serum (10 μL) onto pre-punched filter paper discs. The authors highlight that this method involves a controlled loading procedure, reducing pre-analytical variability.

Notably, several studies have explored the metabolome of dried plasma spots immobilized on cellulose substrates (e.g., [62,63]) and lyophilized plasma (e.g., [64]). The limited adoption of DPS/DSeS in proteomics may stem from the inherent simplicity of the sampling method, which eliminates the need for centrifugation and specialized medical personnel. However, the growing interest in DPS/DSeS within the metabolomics community suggests that proteomic applications may follow, particularly given the translational potential of this approach.

Future research should prioritize advanced sampling media involving additional components, such as supplementary filters or integrated systems designed to isolate functional constituents from whole blood samples more effectively [50,55,65]. For example, to optimize the workflow, it is advisable to begin sample preparation already at the collection stage, which will significantly speed up analysis and improve diagnosis. The concept of intelligent sampling offers a way to reduce sample processing time and improve diagnostic quality compared to the traditional DBS analysis method [4].

One option to accelerate the sample preparation process is to use trypsin to digest proteins on paper [66]. Despite the clear advantages, further study is needed to examine factors such as enzyme availability and peptide extraction efficiency. Another option to speed up analysis is affinity capture and sample purification, which helps work with target proteins at low concentrations [67]. The use of monoclonal antibodies immobilized on cellulose also significantly improves the analysis. Methods have also been developed that allow for the detection of proteins using DBS, utilizing streptavidin and biotin, which provides laboratories with flexibility in configuring the analysis [68].

To reduce the time required to collect specific blood elements, devices such as HemaSpot HF and SE have been developed, which combine collection and storage with an integrated desiccant and serum separation [69]. For example, HemaSpot was used for HIV-1 pol resistance testing in 30 fresh blood samples from the U.S. and 54 previously frozen blood samples from Kenya, with successful genotyping in 79% and 58% of samples, respectively, showing improved results with shorter storage and higher viral load [70].

The VAMS method we mentioned earlier (see Section 1) is also actively used. This method addresses the main issues of DBS, such as the impact of hematocrit, humidity, and volume inaccuracies [71,72]. Using a device with an absorbent polymer tip, VAMS allows precise collection of a fixed blood volume, making the method convenient and practical for pharmacokinetic studies and clinical monitoring.

These innovations may reconcile the theoretical advantages of dried plasma/serum with the practical demands of clinical and field-based applications, potentially bridging the divide between methodological promise and real-world feasibility.

### 2.2. Saliva

The saliva proteome is a promising area for non-invasive diagnostics and clinical applications. Its unique composition and availability make it a valuable biofluid for the detection of both oral and systemic diseases [73]. However, saliva has some significant differences from classical biomaterials, such as blood components related to the activity of proteolytic enzymes and the nonsterile environment of the oral cavity.

Saliva has significant proteolytic activity that can degrade saliva proteins as early as 30 min after sample collection [74], and even protease inhibitors cannot completely stop protein degradation [75]. The use of dry saliva spots (DSaS) may be an answer to this problem. Experimental results comparing proteins in wet saliva and DSaS show that the degradation of proteins dried on filter paper is suppressed in the dry state [76]. As reported in the original study, approximately 2000 salivary proteins (90% of protein identification) exhibited variability within a 0.5- to 2-fold change range (a threshold the authors classified as “relatively stable”), while ~1500 proteins fell within a narrower 0.67- to 1.5-fold change range. Notably, the most variable proteins included hemoglobin subunits, keratin (encoded by KRT15), and aquaporin (encoded by AQP5)—essential for saliva production.

Depending on the research objectives, various types of paper are used for analysis with DSaS. The Whatman FTA™ DMPK-C card is used when protein preservation is required as it does not contain chemical reagents that can denature proteins, unlike DMPK-A and DMPK-B cards, which lyse cells and denature proteins on contact.

However, working with saliva presents a challenge—due to its colorless nature, the sample spot is difficult to identify on such cards visually. Color-indicated cards are used to address this, allowing analysts to easily monitor the placement of the dried sample [77]. Another option is Whatman™ 903 (Whatman, Little Chalfon, England), a filter paper that protects proteins in biological samples. Fixing DSaS on this paper, researchers developed a quantitative method for determining matrix metalloproteinase-1 (MMP1), one of the most promising salivary biomarkers for detecting oral squamous cell carcinoma (OSCC). The developed workflow showed good accuracy (intraday and interday variations < 10%) and precision (80–100%) for the quantification of MMP1 in DSS samples, with a limit of quantification of 3.07 ng/mL [78].

Saliva is increasingly being analyzed in proteomic studies using DSaS methodology, although it remains less well-studied than DBS. Current proteomic methods allow for the determination of up to 5000 proteins per DSaS, many of which are unique to saliva [76]. In addition, DSaS can be used to map the distribution of saliva proteins originating from parotid, submandibular, and sublingual salivary glands; detect significant differences in protein abundance; and identify potential biomarkers for diseases such as Sjögren’s syndrome among proteins specifically expressed in these glands [79].

### 2.3. Tears

The human tear proteome exhibits remarkable complexity, encompassing over 1500 distinct proteins [80]. Tears are a reservoir of potential biomarkers for assessing ocular surface health, inflammatory processes, and lacrimal gland function. Beyond ophthalmic applications, emerging evidence underscores the diagnostic utility of tear proteomics in systemic conditions, including cancers and neurological disorders [81,82,83,84,85,86].

While liquid tear collection in capillary tubes is still common (with occasional mentions of ophthalmic sponges being used [87]), dried tear samples (collected on Schirmer’s strips—filter paper placed under the lower eyelid) are gaining popularity in both clinical and research contexts. The published evidence indicates that Schirmer’s strips provide more detectable proteins and a proportionally higher number of proteins of intracellular origin than the capillary method of tear collection [88]. Moreover, patients generally prefer their use over capillary tubes as dry carriers are safer and perceived as less invasive [89].

A significant advancement in tear sample preservation was reported in 2017, introducing a novel dry storage protocol. Traditional “wet” methods require the immediate flash-freezing of Schirmer’s strips at −80 °C post-collection. In contrast, the dry method involves dehydrating strips using a hairdryer, followed by vacuum sealing using a kitchen sealer [90]. As Qin et al. have noted, the key advantage of the dry method lies in the preservation of proteins through the drying of enzymes such as hydrolases, which prevents protein degradation and enables stable storage at room temperature in vacuum-sealed packages. This innovation enhances logistical feasibility and preserves proteomic integrity, making it particularly advantageous for large-scale studies and paving the way for establishing tear biobanks [90]. In contrast to earlier optimistic assessments of tear fluid biomarker potential, a recent investigation has critically evaluated pre-analytical variability in tear protein analysis [91]. The study highlights unresolved bottlenecks in sample handling, notably observing that the storage of tear specimens as dried strips—without freezing—remains methodologically unvalidated. Comparative analyses revealed that “wet” storage in phosphate-buffered saline better preserves the native proteomic profile than “dry” methods. The authors further underscore a critical gap in the field: despite growing interest in tear-derived biomarkers, standardized protocols for collection, storage, and processing remain conspicuously absent.

Integrating cellulose-based substrates, such as Schirmer’s strips, into large-scale or remote clinical studies introduces additional risks. Proteomic distortions arise from substrate-specific interactions: capillary-tube-collected samples exhibit enrichment of the extracellular proteins linked to immune pathways, whereas Schirmer’s strips yield higher intracellular protein content (e.g., heat-shock proteins, annexins, and S100 family members). These discrepancies may stem from direct conjunctival contact during strip application, which can induce reflex tearing—diluting protein concentrations—and microtrauma to the ocular surface vasculature [92]. The primary sources of contamination when using Schirmer’s strips are the healthcare worker’s gloves or the skin of the tear donor. While contamination from gloves can be eliminated, direct contamination from the patient’s skin is impossible to avoid.

Schirmer’s strips can also be contaminated with analytes not directly related to the object being studied, particularly keratins from the environment [93] and intracellular components [94]. A standard solution is to accompany the target sample applied to Schirmer’s paper with blank Schirmer’s paper controls—this allows for the subtraction of any pre-existing proteins [95]. Such collection-induced artifacts emphasize the need for rigorous methodological harmonization to ensure reproducible biomarker discovery.

### 2.4. Urine

Urine has emerged as a highly viable biofluid for proteomic investigations owing to its minimally invasive collection procedures and established utility in clinical diagnostics [96]. Compared to capillary blood sampling, urine offers unparalleled accessibility, positioning it as the second most frequently analyzed biofluid in routine clinical practice. Its applications span the monitoring and management of conditions such as diabetes mellitus, renal pathologies, urinary tract infections, and other systemic disorders. A landmark example of its diagnostic relevance is the detection of human chorionic gonadotropin (hCG) for pregnancy confirmation, which remains one of the most widely utilized point-of-care assays in global healthcare [97].

Significant progress has been achieved by applying modern proteomic methodologies to identify and characterize protein-based biomarkers for urological cancers. The most prominent example is the prostate-specific antigen (PSA), which has been extensively integrated into clinical practice for monitoring prostate cancer. Bladder tumor antigen (BTA, including BTA Stat^®^ and BTA TRAK^®^ [98]) assays are utilized for bladder cancer detection, often as adjuncts to cystoscopy. For longitudinal monitoring of patients with a history of bladder cancer, another protein-based biomarker—Nuclear Matrix Protein 22 (NMP22, BladderChek^®^ [99])—is employed. Each of these solutions, however, is not exempt from the common limitations inherent to protein biomarkers, which are frequently characterized by moderate specificity that can compromise diagnostic accuracy. The existing biomarkers usually are not employed as standalone diagnostic tools due to the risks of overdiagnosis and false-positive results. Beyond the aforementioned FDA-approved assays, several highly promising candidates are under investigation. These include molecules undergoing clinical validation or currently used in select clinical contexts but lacking universal adoption for routine application (e.g., NGAL, KIM-1, IL-18, and cystatin C).

The composition of urine proteome is less complex than that of serum or plasma, making it more feasible for proteomic analyses where abundant proteins obscure signals derived from less-abundant proteins. On the other hand, dilution introduces variability in urinary protein levels. Additionally, significant fluctuations in pH, urea, and salt concentrations further complicate subsequent analysis and the identification of clinically relevant biomarkers. Other intrinsic factors such as diet, physical activities, and time of collection can also affect the proteome [100].

Despite inherent limitations, urine persists as a cornerstone biofluid in biomarker discovery. Among the so-called urinome of healthy individuals, 70% of proteins originate in the urinary tract, while the remaining are filtered from plasma [96]. Thus, the analysis of urinary proteins as biomarkers is of great importance for urinary tract diseases and diseases affecting more distant organs. A recent study using high-resolution mass spectrometry in tandem with fractionation reports the identification of more than three thousand proteins [101].

Although urine has become an attractive biological fluid for proteomic studies in a broad sense [102], there is a notable lack of experimental studies specifically focused on the proteomic analysis of dried urine samples (e.g., in the form of dried urine spots or dried urine strips). While dried urine spots have been used for metabolomics and small-molecule analysis, such as mycotoxins [103] and hormones [104], their application in proteomics is still in its infancy. As a singular example of a proteomic study utilizing dried urine spots (DUSs) documented in the literature, the methodological approach to urine preparation merits particular attention. This technique is achieved by integrating a 5-milliliter medical syringe and a protein-adsorbent membrane, facilitating efficient sample processing and stabilization for subsequent proteomic analysis. Syringe-push nitrocellulose membrane absorption (SPMA) of urine provided the greatest quality of proteome profile as demonstrated by 2DE and overperformed three current methods of urine preparation (i.e., ultrafiltration, dialysis/lyophilization, and precipitation). Proteins absorbed on nitrocellulose harvested from the SPMA procedure could be stored as a dried membrane at room temperature for at least 1 month without noticeable protein degradation [105].

The proteome has been the least explored among the various studies conducted on dry urine samples [106]. This gap in the literature points to a significant opportunity for future research to explore whether dried urine matrices can sufficiently preserve protein integrity and complexity to support reliable proteomic biomarkers and clinical diagnostics. We hope that with developed volumetric technologies and normalization approaches, the area of the dried urine proteome can be explored effectively.

## 3. Considerations on Sample Preparation and Analysis Techniques

With the great advantages of sample collection and storage, challenges associated with sample preparation and analysis of dried samples come, especially in mass-spectrometry-based approaches. Aside from the biofluid type, protein extraction is the first step in the preparation pipeline. Extraction mixture composition selection depends on the properties of target proteins; however, common mixtures used for profiling purposes often include buffers for pH stability during downstream digestion procedures with the addition of detergents and chaotropic agents to perform remaining cell lysis and improve protein solubilization and denaturation.

In the case of blood-related samples, ammonium bicarbonate buffer (ABC) [26,107] is commonly used for further bottom-up experiment processing. At the same time, applying ELISA and other immunoassays is often associated with using phosphate-buffered saline (PBS) [69,108,109] due to aspects of those approaches. Immunoassays commonly utilize the addition of detergents for both solubilization and protein–protein interaction improvements. However, protocols depend on the actual detecting system being used: Omosule et al. [110] reported that in the case of the detection of SARS-CoV-2 antibodies, utilization of detergent-free PBS and water provides a lower background signal. Whittaker et al. [111] reported similar results: while the number of proteins detectable with the antibody array was not much lower when removing the detergent, observed intensities were higher than Tween samples.

On the mass-spectrometry-based proteomics side, procedures are predictably more complicated. While using detergents eases the extraction process, part of the work utilizes simple buffer-only extraction systems [112,113,114] as most of the available detergents are not MS-compatible and require removal before analysis before removing unwanted signal interference. However, the utilization of detergents in combination with further phase extraction purification techniques is quite effective: Guedes et al. [115] reported the detection of over 400 protein groups, corresponding to 4000 peptides—with roughly 25% exhibiting PTM—in DBS from five coronary artery disease patients and five controls. The proteins were extracted using a mixture of ABC, sodium deoxycholate, and SDS before being processed with the SP3 protocol [116]. Kashirina et al. [107,117] suggest adding reducing agents, such as TCEP, to the extraction buffer to improve denaturation. The authors reported that 1300 proteins were quantified. On the other hand, there is a variety of MS-compatible detergents. The utilization of commercially available RapidGest is described in the context of target-proteomics studies by Refat et al. [118] and Sumaily et al. [119]. Similarly, Protease Max was used during immuno-SRM assay development [120]. As mentioned, studies have described pricy approaches but the additional usage of non-low-cost detergents looks predictable. When analyzing large cohorts, more cost-efficient solutions should be used—e.g., sodium laurate, which is suitable for membrane protein solubilization and easily removed from the mixture before analysis [121].

Selective extraction of target proteins and the depletion of major constituents are standard procedures in blood proteomics [122]. However, there are no studies where workflows with depletion resins are utilized in DBS analysis, possibly due to its high cost. Alternative approaches have been suggested by Nakajima et al. [17]. The authors discussed sodium bicarbonate solutions for selective solubilization of the major hydrophilic blood constituents and precipitation of other blood proteins. Compared to sodium laurate mentioned above [123], the number of quantified proteins increased from 521 to 990. However, hydrophilic proteins have not been removed completely so if any information on their modifications should be gained, this approach suits this task. Despite protein recovery issues, this method is one of the most effective in terms of both information and cost among the abovementioned approaches. It should also be noted that other techniques of non-affinity blood sample depletion—such as chloric acid-based precipitation—demonstrated efficiency in large cohort studies [124], and it would be interesting to see its application to DBS samples.

There is a lack of MS-based proteomics studies related to other biofluids, which, as mentioned earlier, are likely to be associated with the specifics of such sample types. Besides DBS studies, one interesting work has been published recently dealing with DSaS [76]. Sato et al. compared whole saliva profiling with DSeS and DBS. Samples were extracted with 1% SDS and purified using the SP3 pipeline. The authors reported that about 5000 proteins were quantified in both whole saliva and DSeS samples compared to 2000 in DBS samples, which mostly overlapped the saliva samples profile, demonstrating that DSeS is a very informative addition to DBS samples.

Another critical factor in the experiment design of untargeted studies is the choice of a suitable proteome profiling approach.

There are still no significant hardware differences between data acquisition approaches (meaning data-dependent and data-independent techniques [125]) in analyzing complex mixtures such as cell lines lysates, especially in samples with a wide range of protein concentrations and highly abundant components such as blood. In DDA (data-dependent acquisition), the mass spectrometer first performs a full survey scan to detect ions in the sample, and then automatically selects the most abundant ions for fragmentation and further MS/MS analysis. This approach prioritizes high-intensity signals, making it effective for identifying well-expressed proteins. However, it often misses low-abundance peptides as they are not selected for fragmentation. In contrast, DIA (data-independent acquisition) fragments all ions within predefined mass-to-charge (m/z) windows, regardless of their intensity. Instead of targeting specific peaks, the instrument systematically divides the entire m/z range into small windows and fragments all ions within each window. This ensures that even low-abundance peptides are captured, providing a more comprehensive view of the proteome. However, the resulting MS/MS spectra are highly complex as they contain fragments from multiple co-eluting peptides. Analyzing DIA data requires advanced bioinformatics tools (e.g., spectral libraries or machine learning algorithms) to deconvolute these mixed spectra. DIA is particularly valued for quantitative studies, such as comparing protein expression across large sample cohorts, due to its high reproducibility and reduced missing-data issues. DIA outperformed [126] DDA due to the lower impact of stochastic factors on the results and more precise quantification. Blood and its components exhibit an extremely wide dynamic range (spanning 12 orders of magnitude [127]), making DIA a more attractive technology for obtaining unbiased spectra. This approach enables complete, balanced profiling of the blood proteome without skewing toward highly abundant proteins like albumin and macroglobulin. Therefore, similarly, in the case of DBS, DIA demonstrated a higher number of identifications/quantifications per run [123]. Another significant improvement could be achieved with additional ion-mobility separation of the analyzed peptides mixture. Several papers [128,129] demonstrated the efficiency of the High-Field Asymmetric Waveform Ion Mobility (FAIMS) usage in cases of DBS analysis, showing the number of identified proteins doubling.

In conclusion, while extensive research has been conducted on proteomic analysis of blood-related samples, studying other biofluids in their preserved, dried spot form requires further investigation. Moreover, despite the availability of cost-effective depletion techniques and a variety of mass-spectrometry-compatible sample preparation tools, a standardized pipeline for processing such samples has yet to be established. The development of standardized protocols is crucial, particularly for clinical applications where consistency and reproducibility are essential. Nevertheless, given that dried spot samples can yield as much information as native biofluids, they represent a promising and viable alternative to conventional liquid biopsy approaches.

## 4. Prospects

The clinical adoption of dried biofluid spots is driven by their unique practical advantages: simplified logistics (room-temperature storage and transport), reduced contamination risks, and patient self-collection feasibility. These features address critical barriers in low-resource settings, pediatric care, and large-scale screening programs where conventional liquid sampling is impractical.

Our analysis reveals a pronounced disparity in dried biofluid research, with most studies focused on blood proteomics and its components. This bias is pragmatically justified: blood remains the clinical “gold standard” for disease diagnosis, monitoring, and detection of illicit substances.

In the pursuit of spatially relevant biomarkers, tissue-adjacent biofluids (e.g., tears and saliva) offer a compelling intermediate between proximal (organ-specific) and distal (systemic) profiling [25,130,131]. These fluids combine the accessibility of peripheral samples with molecular signatures enriched for tissue-specific pathways, enabling both simplified collection and enhanced biological relevance for disease-specific studies. To provide a comprehensive landscape of current research, we further reviewed the literature regarding various dried biofluids—including urine, tears, saliva, plasma, and serum—across different omics levels.

**Figure 2:**
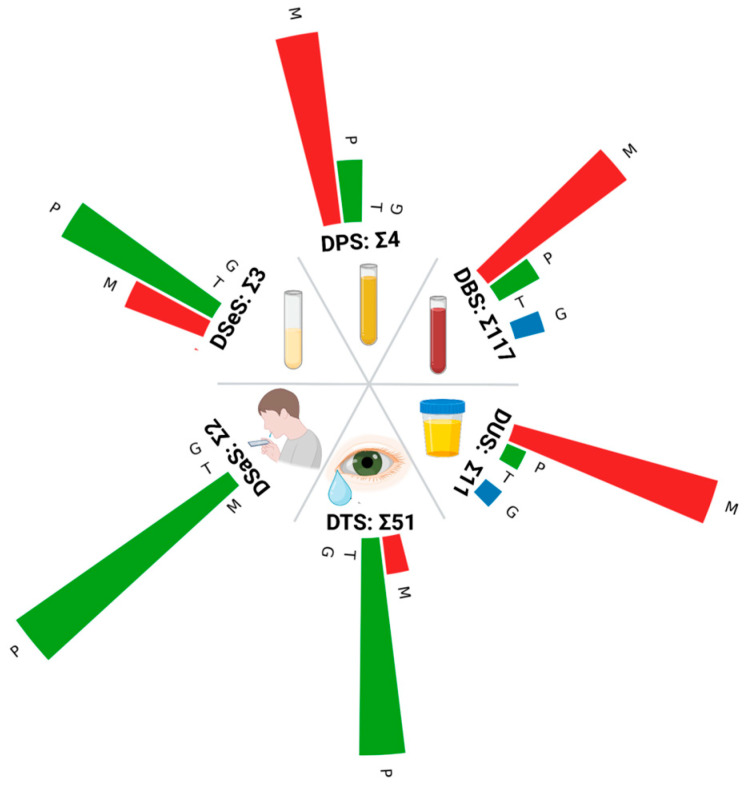
Number of articles in PubMed for each type of biological liquid discussed: G—genomics (blue); T—transcriptomics (yellow); P—proteomics (green); M—metabolomics (red). ∑ indicates the summarized number of articles for each type of biological sample. The search in PubMed was effected using the following queries: for DBS: ((“dried blood spot”[Title/Abstract]) OR (“dried bloodspot”[Title/Abstract])) AND (OMICS[Title/Abstract]); DPS: (“Dried Plasma Spot”[Title/Abstract]) AND (OMICS[Title/Abstract]); DSeS: (“Dried Serum Spot”[Title/Abstract]) AND (OMICS[Title/Abstract]); DTS: ((Schirmer’s Strip)[Title/Abstract] OR (“Dried Tear Strip”)[Title/Abstract]) AND (OMICS[Title/Abstract]); DUS: ((“Urine Strip”)[Title/Abstract] OR (“Dried Urine Strip”)[Title/Abstract] OR (“Dried Urine Spot”)[Title/Abstract]) AND (OMICS[Title/Abstract]); DSaS: (“Dried Saliva Spot”[Title/Abstract]) AND (OMICS[Title/Abstract]).

Despite the widespread adoption of DBS and DTS, other biofluids remain underexplored in proteomic contexts. This gap is particularly striking given the availability of metabolomic data from dried urine samples, which suggests the technical and methodological feasibility of such studies (Figure 2) [132,133]. While biofluids hold significant promise for non-invasive biomarker discovery, each presents distinct analytical challenges. Urine exhibits marked variability in protein concentration due to hydration-dependent dilution effects [134], while the salivary proteome is confounded by dynamic shifts in the oral microbiome [135]. Reproducibility issues found in the proteomics of tear fluid and variability found in urine and saliva highlight the growing challenges in the field, which is at the stage of transitioning from theoretical promises to practical applications. To visualize the inequality in biofluid research using different omics approaches, we used the number of articles in PubMed (Figure 2). It is worth noting that for most of the biofluids, more detailed searching reveals even more articles as authors do not always mention the omics technology in the Mesh Term or Title/Abstract. For instance, for DSaS, we additionally found at least three articles researching the metabolome [136,137,138].

Despite the maturity of genomics and transcriptomics, studies encompassing all biofluids across these omics layers remain relatively scarce (Figure 2). The paucity of transcriptomic investigations can be primarily attributed to RNA instability and suboptimal preservation conditions compared to DNA, proteins, and metabolites. Conversely, in genomics, the predominant barrier remains the considerable cost associated with sequencing technologies, highlighting the imperative for cost-reduction strategies. Concurrently, increasing efforts to integrate proteomics and metabolomics into clinical practice have substantially driven research activity in these specific omics levels.

DBS emerges as the most extensively studied dried biofluid, reflecting robust scientific community interest across genomic, transcriptomic, proteomic, and metabolomic platforms (Figure 2). DTS follows as the second-most investigated biofluid, primarily within proteomic and metabolomic contexts. Nevertheless, proteomic analyses of tear fluid encounter significant challenges due to reproducibility concerns arising from inconsistent collection methodologies [139]. Employing targeted analytical approaches could address some of these methodological constraints, bolstering the potential of other biofluids as complementary reservoirs of biomarkers. However, inherent biological and methodological variability emphasizes the critical and immediate need for standardized, reproducible protocols.

Expanding research to alternative biofluids and multi-omics integration could unlock transformative potential for non-invasive diagnostics, longitudinal cohorts, and global biobanking initiatives, where the logistical advantages of dried samples—ease of collection, storage, and transport—are paramount [15,140]. Key barriers include protein degradation risks, contamination, and a lack of standardized protocols. However, lessons from DBS development provide a foundational framework for universal solutions. Future efforts must prioritize biomarker validation in alternative biofluids, analytical optimization, and international protocol consensus. Such advances could transition dried samples from niche tools into scalable platforms for large-scale, representative, and minimally invasive research, aligning with the demands of precision medicine.

Theoretically, dried biofluids could underpin planetary-scale proteomics initiatives. Their non-invasive nature, long-term stability, and low-cost logistics enable mass proteome profiling across diverse populations. By capturing real-time physiological states in a standardized format, these samples could democratize access to biomarker discovery, fostering equitable advancements in global health diagnostics.

## Abbreviations

The following abbreviations are used in this manuscript:

DBS	Dried blood spot
DSeS	Dried serum spot
DSaS	Dried saliva spot
DDA	Data-dependent proteomic analysis
DIA	Data-independent proteomic analysis
FDA	Food and Drug Administration
PKU	Phenylketonuria
SPMA	Syringe-push nitrocellulose membrane absorption
VAMS	Volumetric absorptive microsamplers
